# Effects of Revascularization on Cardiac Autonomic Neuropathy in Type 2 Diabetes Mellitus Patients With Chronic Stable Angina

**DOI:** 10.7759/cureus.72278

**Published:** 2024-10-24

**Authors:** Waqas Alauddin, Prajakta M Radke, Pooja Pardhi, Mohit Mishra, Shashwat Arora

**Affiliations:** 1 Physiology, Naraina Medical College and Research Center, Kanpur, IND; 2 Physiology, MGM Medical College, Navi Mumbai, IND; 3 Internal Medicine, MGM Medical College, Navi Mumbai, IND; 4 Medicine, Naraina Medical College and Research Center, Kanpur, IND

**Keywords:** cardiac autonomic neuropathy, cardiovascular reflex tests, chronic stable angina, heart rate variability (hrv), parasympathetic tone, percutaneous coronary intervention (pci), sympathetic tone, type 2 diabetes mellitus

## Abstract

Background: Type 2 diabetes mellitus (T2DM) leads to cardiac autonomic neuropathy (CAN), affecting blood flow and heart rate regulation eventually causing chronic stable angina (CSA). Percutaneous coronary intervention (PCI) can correct autonomic dysfunctions and improve myocardial perfusion. This study aimed to assess CAN using heart rate variability (HRV) and cardiovascular reflex tests in T2DM patients with CSA before and after PCI.

Material and methods: From cardiology outpatient clinics, 30 patients with T2DM with CSA were recruited. Before and after PCI, the following HRV parameters like low frequency (LF), high frequency (HF), LF:HF ratio, total power (TP), square root of mean square differences of successive R-R intervals (RMSSD), standard deviation of normal R-R intervals (SDNN), and percentage of adjacent NN intervals that differ from each other by more than 50 seconds (pNN50) were measured for every patient. Cardiovascular reflex tests, including the deep breathing test, the isometric handgrip test, the 30:15 ratio, the Valsalva ratio, and the lying-to-standing test (LST), were performed on the patients both before and after PCI. SPSS version 21.0 (IBM Corp., Armonk, NY), a licensed statistical program, was used to compile and analyze the data.

Results: When compared to the pre-PCI group, 30 patients with T2DM with CSA, age range between 45 and 70 years, both genders demonstrated a significant increase in post-PCI HRV frequency domain parameters, such as LF (239.52±67.21ms^2^ vs. 307.62±74.17 ms^2^) and HF (249±25.21 ms^2^ vs. 379.67±76.55 ms^2^). The time domain parameters showed a significant increase in post-PCI values compared to pre-PCI values. These included RMSSD (33.97±3.96 ms vs. 8.005±3.25 ms), SDNN (41.4±9.78 vs. 31.74±7.04ms), NN50 (13.241±3.07 vs. 5.20±6.63), and TP (1130.08±320.10 ms^2^ vs. 754.54±96.93 ms^2^). In cardiovascular reflex tests, pre-PCI groups had significantly lower delta HR (DBT) (10.47±1.76 bpm vs. 14.20±2.09), E:I ratio (DBT) (1.14±0.042 vs. 1.20±0.026), and Valsalva ratio (1.10±0.071 vs. 1.22±0.084) than post-PCI groups. The pre-PCI group showed a substantial decrease in both the systolic blood pressure (LST) (-6.13±4.85 mmHg vs. -1.01±3.63 mmHg) and the 30:15 ratio (1.13±0.074 vs. 1.09±0.067) when compared to the post-PCI group. The handgrip test (18.73±4.31 mmHg vs. 15.31±4.27 mmHg) did not yield statistically significant results.

Conclusions: Before PCI, T2DM patients with CSA experienced autonomic dysfunction, but after revascularization, their functions improved by reestablishing basal cardiac autonomic tone and autonomic reactivity. HRV and cardiovascular reflex tests are useful noninvasive tools for assessing CAN and stratifying a prospective risk factor for estimating the morbidity and death from cardiovascular illnesses in T2DM with CSA.

## Introduction

The main cause of the high and increasing type 2 diabetes mellitus (T2DM) burden globally, especially in middle-income nations like India, is the prevalence of overweight and obesity as well as unhealthy lifestyles [1]. According to the latest WHO estimates, over 77 million people over the age of 18 have T2DM, with nearly 25 million prediabetics [1].

Cardiac autonomic neuropathy (CAN) is a significant consequence that is typically caused by an underlying disease, such as diabetes [2]. It has a detrimental effect on the autonomic nervous system and cardiac function regulation, resulting in heart rate control and blood flow dysfunction [2]. It is usually caused by an imbalance in the cardiovascular system's sympathetic and parasympathetic nervous systems [3]. Hyperglycemia, oxidative stress, and inflammation are common causes of this phenomenon. Moreover, CAN affects multiple organ systems, which makes it a primary cause of morbidity and mortality in patients with diabetes [1]. Glycemic management, systolic and diastolic blood pressure and aging-related neuronal loss can all interact intricately [1]. Patients may have resting tachycardia, orthostatic hypotension, and a silent myocardial infarction [3]. CAN is typically seen in patients with long-term, uncontrolled diabetes [3,4]. Due to insulin resistance and related metabolic disorders, T2DM accelerates atherosclerosis [3,4]. This can result in endothelial damage, reduced vasodilation, and increased plaque formation, all of which can hasten the development of coronary artery disease (CAD) [3,4].

One kind of chest pain or discomfort in CAD is called chronic stable angina (CSA) [5]. Patients with T2DM are more likely to have central obesity and dyslipidemias, as well as a sedentary lifestyle causing atherosclerosis, which increases the prevalence of CSA and is also associated with autonomic dysfunctions [3]. CSA can lead to myocardial infarction or other more serious forms of CAD, eventually causing death [5]. Over time, recurrent ischemia can damage the heart muscle and cause cardiac failure. It carries a high mortality risk and has serious consequences if improperly handled [5].

Percutaneous coronary intervention (PCI) may be required in some situations to enhance blood flow and lower the risk of unfavorable, severe, and life-threatening complications [5]. The chosen revascularization strategy depends on the patient's comorbidities and the location and severity of the blockages [5]. Thus, improving the myocardial perfusion by revascularization procedures like PCI will rectify autonomic dysfunctions [5]. As far as we are aware, this shall be the first research to examine the impact of PCI therapy-induced revascularization on CAN and to perform cardiovascular reflex tests and heart rate variability (HRV) in patients of T2DM with CSA. The purpose of the current study was to determine if patients with T2DM who had undergone PCI showed improvements in their CAN.

## Materials and methods

This is pre- and post-interventional research. This study, which was conducted in association with the section of cardiology along with the Department of Physiology, was approved by the Institutional Ethics Committee of Vardhman Mahavir Medical College (VMMC) and Safdarjung Hospital in New Delhi. It was conducted from November 2017 to April 2019. The approval number for this research is IEC/VMMC/SJH/Thesis/October/2017-177. This study included thirty people with T2DM and CSA, ages 45 to 70. Any gender could be one of the subjects. They were selected from Safdarjung Hospital's and VMMC's cardiology outpatient patient lists. Before and after these subjects' main coronary arteries underwent six months of PCI, after obtaining written consent, cardiovascular reflex tests and HRV readings were obtained. All autoimmune diseases, collagen diseases, congenital heart disease, arrhythmias, valvular heart disease, psychiatric conditions, uncontrolled high blood pressure and DM, congestive heart failure, and other medical comorbidities with a percentage of ejection fraction less than 35% were not allowed to participate in the study.

Prior to and following PCI, baseline measurements such as heart rate and systolic and diastolic blood pressure, as well as BMI, HbA1c, fasting, and postprandial blood glucose, were taken. The analysis of HRV was done as participants were advised to lay in the supine position on the actual day of the test. The electrodes were inserted into lead II and were instructed to rest for 10 to 15 minutes. The electrocardiogram (ECG) was recorded for five minutes, during which time the subjects were directed not to move, speak, cough, or sleep. Conventional frequency-domain measurements were employed to measure the power spectrum, and the time and frequency domain analysis was conducted using the fast Fourier transform (FFT) [5].

The cardiovascular reflex tests included the handgrip test, deep breathing exercises, cold pressor test, Valsalva maneuver, and lying-to-stand test (LST) to determine the effect of cardiovascular autonomic reactivity. Participants were told not to eat or drink caffeine for four hours before the experiment in order to reduce confusion [6].

Test of deep breathing

Six gentle breaths per minute were to be inhaled and exhaled by the participants after a five-minute rest period. Throughout this deep breathing exercise, an ongoing ECG recording was made. The next was the calculation of the ratio of expiration to inspiration (E:I). The mean of the shortest and longest RR intervals throughout expiration and inhalation is known as the E:I ratio. Then the heart rate delta was noted [6].

The Valsalva maneuver involved the participants forcefully exhaling through a mouthpiece attached to a sphygmomanometer and holding an expiration pressure for 15 seconds at 40 mmHg. At the same time, lead II's ECG had been recorded for 60 seconds during both periods of relaxation and forceful exhalation. The Valsalva ratio (VR) was calculated as the proportion of the maximum Phase IV RR interval relative to the minimum Phase II RR interval [6].

The ratio of standing to lying (30:15)

This test assessed the change in heart rate when shifting from supine to a position of standing. The subjects were tilted 60 degrees with a simple tilt table and then remained there for five more minutes. The 30:15 ratio is the proportion of the minimum RR interval, which occurs roughly on the 15th heartbeat following standing, to the maximum RR interval, which occurs on the 31st heartbeat after standing [6].

Test of isometric handgrip

Using a mercury sphygmomanometer, baseline blood pressure was determined following a five-minute rest period. Subjects were directed to use their dominant hand and all of their force while using the dynamometer in their hand for a short while in order to obtain the maximum voluntary contraction. After that, the highest force used was noted. The participants were then instructed to apply for the duration of four minutes of force to the dynamometer at 30% of their maximum muscular contraction. Additional blood pressure readings were taken at one, two, and four minutes. An initial measurement of ΔDBP was taken [6].

Both parasympathetic and sympathetic nervous system activity are measured during this exam, which goes from lying down to standing. In order to gauge sympathetic activity, SBP was measured both while lying down and at the passive tilt. Blood pressure readings were taken at 30, one, two, and five minutes [6].

Data entry and analysis

The data were entered and examined using the statistical software SPSS version 21.0 (IBM Corp., Armonk, NY), which was licensed. Mean±standard deviation (SD) was the data presentation format. Pairwise student t-tests were used to determine the statistical value of the variations among the pre- and post-interventions. A p<0.05 significance level was applied.

## Results

Thirty patients of T2DM with CSA who were undergoing PCI between the ages of 45 and 70 years, their baseline parameters (pre-PCI) and the post-PCI values were taken after six months to emphasize the follow-up duration.

Basal characteristics of T2DM patients with CSA

Table 1 shows that the age of the T2DM with CSA patients was 53.7±8.9 years, and their body mass index was found to be 24.23±2.59 kg/m2. HbA1c was 8.24±0.67 in pre-PCI T2DM with CSA and 7.82±0.65 in post-PCI T2DM with CSA.

**Table 1:** Basal characteristics and biochemical parameters in T2DM patients with CSA before and after PCI *p-value less than 0.05 is statistically significant. The values are expressed as mean ± SD; SD: Standard deviation; HbA1c - hemoglobin A1c; SBP - systolic blood pressure; DBP - diastolic blood pressure; HR - heart rate; mg/dL - milligram per deciliter of blood; mmHg - millimeters of mercury; bpm - beats per minute; T2DM - type 2 diabetes mellitus; CSA - chronic stable angina; PCI - percutaneous coronary intervention

Parameters	Pre-PCI type-2 DM with CSA (n=30), Mean±SD	Post-PCI type-2 DM with CSA (n=30), Mean±SD (after six months)	P-value
Age (years)	53.7±8.9	54.0±8.9	NS
BMI	24.23±2.59	24.58±2.71	NS
HbA1c	8.24±0.67	7.82±0.65	0.01*
Fasting blood glucose (mg/dL)	156.80±13.49	140.57±10.19	<0.000*
Postprandial blood glucose (mg/dL)	266.73±25.59	237.93±21.57	<0.000*
Systolic blood pressure (mmHg)	149.40±17.6	121.47±9.80	<0.000*
Diastolic blood pressure (mmHg)	91.23±11.27	76.90±8.16	<0.000*
Heart rate (bpm)	86.87±12.77	76.87±5.75	<0.000*

HRV in T2DM patients with CSA

Table 2 presents the time-domain parameters of SDNN (41.4±9.78 vs. 31.74±7.04 ms, p<0.0001), RMSSD (33.97±3.96 ms vs. 8.005±3.25 ms, p=0.0001), and pNN50 (13.241±3.07 vs. 5.20±6.63, p<0.0001), which were all significantly higher when comparing the post-PCI values compared to pre-PCI readings. There had been substantial rises in both the HF (379.67±76.55 ms^2^ vs. 249.35±25.21 ms^2^, p<0.0001) and LF (307.62±74.17 ms^2^ vs. 239.52±67.21ms^2^, p<0.001) when comparing the post-PCI values to the pre-PCI values (Table 2). In comparison to pre-PCI, post-PCI total power (TP) increased significantly (1,130.08±320.10 ms^2^ vs. 754.54±96.93 ms^2^, p < 0.00). Its LF/HF proportion was greater in the pre-PCI group (1.86±2.13 vs. 1.38±1.41, p=0.938) than in the post-PCI group, but there was no statistically significant difference between the two.

**Table 2:** Heart rate variability (HRV) – time domain and frequency domain parameter among T2DM patients with CSA before and after PCI *p-value less than 0.05 is statistically significant. The values are expressed as mean ± SD;  SD: Standard deviation; HRV - heart rate variability; SDNN - standard deviation of the normal to normal R-to-R interval; RMSSD - square root of mean squared differences of successive NN intervals; pNN50 - percentage of number of intervals differing by >50ms from the adjacent interval; ms - millisecond; LF - Low frequency; HF - high frequency; LF/HF - Low frequency by high frequency ratio (); millisecond squared (ms2); % - percentage; T2DM - type 2 diabetes mellitus; CSA - chronic stable angina; PCI - percutaneous coronary intervention

HRV parameters	Pre-PCI type-2 DM with CSA (n=30), Mean±SD	Post-PCI type-2 DM with CSA (n=30), Mean±SD (after six months)	P-value
SDNN (ms)	31.74±7.04	41.4±9.78	<0.000*
RMSSD (ms)	26.44±50.2	33.97±3.96	<0.000*
pNN50 (%)	8.005±3.25	13.241±3.07	<0.000*
LF (ms²)	239.52±67.21	307.62±74.17	<0.000*
HF (ms²)	249.35±25.21	379.67±76.55	<0.000*
Total power (ms²)	754.54±96.93	1130.08±320.10	<0.000*
LF:HF	1.86±2.13	1.38±1.41	0.938

Cardiovascular reflex tests in T2DM patients with CSA

Table 3 depicts patients with PCI having a delta heart rate that was significantly higher than patients without the procedure (14.20±2.09 versus 10.47±1.76 bpm, p = 0.000). The E:I ratio was considerably greater among PCI (1.20±0.026 versus 1.14±0.042, p = 0.000) than in the post-PCI group. According to the VR, following PCI, patients had noticeably greater parasympathetic reactivity as compared to the pre-PCI (1.22±0.084 vs. 1.10±0.071, p = 0.00). Patients who had PCI had significantly higher parasympathetic reactivity (1.13±0.074 vs. 1.09±0.067) as measured by the 30:15 ratio (p = 0.001) than patients who did not have PCI. Compared to post-PCI patients, pre-PCI patients had a significantly higher drop in SBP during LST (-6.13±4.85 versus -1.01±3.63, p=0.03). Table 3 also shows that the increase in IHT's DBP was significantly higher (18.73±4.31 mmHg versus 15.31±4.27 mmHg, p=0.113) when comparing post-PCI patients to pre-PCI patients.

**Table 3:** Cardiovascular reflex tests among T2DM patients with CSA before and after PCI *p value less than 0.05 is statistically significant. Values are expressed as mean ± SD; SD: Standard deviation; n - number; ∆ HR - delta heart rate; E:I ratio - expiration/inspiration; LST - lying to standing test; ∆ SBP - delta systolic blood pressure; IHT - isometric handgrip test; ∆ DBP - delta diastolic blood pressure; T2DM - type 2 diabetes mellitus; CSA - chronic stable angina; PCI - percutaneous coronary intervention

Cardiovascular reflex test parameters	Pre-PCI type-2 DM with CSA (n=30), Mean±SD	Post-PCI type-2 DM with CSA (n=30), Mean±SD (after six months)	P-value
∆ HR	10.47±1.76	14.20±2.09	<0.000*
E:I ratio	1.14±0.042	1.20±0.026	<0.000*
Valsalva ratio	1.10±0.071	1.22±0.084	<0.000*
Lying to standing test (LST)-Fall in systolic BP	-6.13±4.85	-1.01±3.63	0.03*
Lying to standing test (LST)- 30:15 ratio	1.09±0.067	1.13±0.074	<0.001*
Isometric handgrip test (IHT)- ∆ DBP	15.31±4.27	18.73±4.31	0.113

## Discussion

This study found that in T2DM patients with CSA, the time-domain variables among pre-PCI, such as pNN50, RMSSD, and SDNN values, were considerably lower than those obtained after PCI. In addition, there was a significant difference between pre-PCI and post-PCI patients in frequency-domain variables like TP, HF, and LF. This suggests a low HRV, citing reduced parasympathetic activity and a disparity between the sympathetic and parasympathetic nervous systems.

The prevalence of diabetes-induced CAN in diabetic patients varies across different studies. A study by Jyotsna et al. found abnormal cardiac autonomic function in 71% of diagnosed diabetic patients, compared to 33.76% of controls [7]. The study's mean HbA1c was 8.03 ± 1.88 g/dL, possibly indicating delayed diagnosis and poor control. Other studies found a prevalence of CAN in 70% of diabetic individuals with an average diabetes duration of 9.03 ± 6.4 years and a mean HbA1c of 10.9 ± 15.5 g/dL [8]. The study's higher CAN prevalence may be due to the faster progression of diabetes in the Indian population, which is associated with increased insulin resistance and a greater risk of micro- and macrovascular complications [9]. Early diagnosis of CAN can prevent the development of comorbidities and provide better longevity without complications [3]. In our study, there was a significant improvement in HbA1c (p=0.01) in the post-PCI group.

Diabetes-related CAN is a complicated condition that is impacted by blood pressure, glycemic control, the length of the disease, and neuronal death due to aging [10]. Hyperglycemia is the leading cause of oxidative stress, toxic glycosylation products, neuronal dysfunction, and death [10]. This is brought on by an increase in free reactive oxygen species production in the mitochondria [11].

Numerous processes, including advanced glycosylation end products, polymerase reductase pathway activation, DNA damage, and detrimental effects on neuronal regeneration and repair, are brought on by diabetes and contribute to neuronopathic changes [4,10,11]. The advancement of CAN may also be facilitated by alterations in the microcirculation, elevated cytokine production, inflammation, genetic predisposition, obstructive sleep apnea, decreased C-peptide levels, and autoimmune antibodies [10]. Diabetes has a length-dependent effect on autonomic nerves, frequently causing symptoms in the vagus nerve, which is responsible for nearly three-quarters of parasympathetic activity [4,11]. These findings also correspond to HRV and cardiovascular reflex tests in pre-PCI values.

Aydinlar et al. found, in line with our research, that following PCI, HF, SDNN, and RMSSD were substantially elevated [12]. However, they noticed an increase in LF and a reduction in the LF:HF ratio [12]. Alauddin et al. found that there was a significant increase in LF, HF, SDNN, and RMSSD after PCI, which were similar to our findings [5]. They also noticed a decrease in LF:HF ratio which signifies improved sympathovagal balance, though it was not statistically significant [5]. A subsequent investigation by Abdelnaby et al. showed that individuals with one artery CAD had an imbalanced sympathovagal system due to malfunction of the zonal section of the left ventricle and that left ventricular dysfunction and HRV were improved by PCI intervention [13]. Similarly, a study by Sedziwy et al. concluded that successful revascularization restored autonomic balance between pre- and post-PCI patients' HRV time-domain variables [14]. Our study also yielded the same results.

By contrasting the effects of deep breathing, the Valsalva maneuver, and sudden change of posture causing changes in the R-R interval (30:15 ratio), the parasympathetic reactivity has been assessed. A decreased parasympathetic reactivity was observed in T2DM with CSA, as evidenced by the VR, 30:15 ratio during the LST, and the E:I ratio with delta HR during the deep breathing test, and all of these were substantially reduced in the pre-PCI period than in the post-PCI period. Our results thus confirm the finding that patients with T2DM with CSA have reduced vagal regulation of their heart rate, which is then restored by PCI revascularization in these individuals [5].

Hence, our findings suggest that pre-PCI patients had autonomic dysfunction, primarily due to the impairment of parasympathetic tone. The equilibrium of both the sympathetic and parasympathetic nervous systems have an impact on the cardiac pacemaker SA node, which is mainly determined by HRV analyses; thus, these autonomic dysfunctions can be rectified by revascularization procedures and also by physical maneuvers and lifestyle changes. Early detection and lifestyle modification are crucial in limiting the negative effects of severe DM-associated CAN.

We suggest carrying out extensive long-term studies with more participants in considering the limitations of this study to get more conclusive results. The study's sample of patients was small. Because the study population was primarily composed of males with a small portion of females, it is imperative to conduct an exclusive study involving females. There should be more research done in this area because very few studies have been done in the Indian community and conflicting results have been found regarding cardiac autonomic functions in T2DM patients undergoing PCI.

## Conclusions

This study emphasizes CAN in patients with T2DM with CSA. These patients had autonomic dysfunction prior to PCI; however, following revascularization, autonomic functions are significantly improved by reestablishing basal cardiac autonomic tone and autonomic reactivity. While HRV and cardiovascular reflex tests are useful tools for assessing autonomic dysfunction, these gold-standard tests provide methods for measuring autonomic functions. These results point to a possible connection between CAN and T2DM in CSA patients. The results of these tests may provide useful information about a prospective risk factor for estimating the morbidity and death from cardiovascular illness in T2DM with CSA. Secondly, more study is necessary to completely understand the effectiveness of these tests and their possible uses in clinical settings. These tests may be used as a non-intrusive screening tool to determine the existence and degree of autonomic disorders in T2DM with CSA.
